# Ursodeoxycholic Acid Prophylaxis and the Reduction of Gallstone Formation After Bariatric Surgery: An Updated Meta-Analysis of Randomized Controlled Trials

**DOI:** 10.7759/cureus.50649

**Published:** 2023-12-17

**Authors:** Mohammad Al-huniti, Yousef Alsardia, Alaa Odeh, Belal Bdour, Ramadan Hassanat, Ali Aloun, Ban W Sha’ban, Sara M Nseirat

**Affiliations:** 1 Department of General Surgery, Jordanian Royal Medical Services, Amman, JOR

**Keywords:** cholecystectomy rates, meta-analysis, ursodeoxycholic acid prophylaxis, bariatric surgery, gallstone formation

## Abstract

Gallstone formation following bariatric surgery poses a significant clinical concern, prompting various preventive strategies, including ursodeoxycholic acid (UDCA) prophylaxis. This systematic review and meta-analysis aimed to assess the efficacy of UDCA in preventing gallstone formation after bariatric surgery.

A comprehensive literature search was conducted in major databases up to September 2023, identifying 12 randomized controlled trials (RCTs) meeting the inclusion criteria. The studies, spanning from 1993 to 2022, involved 2,767 patients who underwent diverse bariatric procedures. The primary outcome was the overall incidence of cholelithiasis, with secondary outcomes encompassing gallstone occurrences at three, six, and 12 months; symptomatic cholelithiasis; and rates of cholecystectomy. The Cochrane risk-of-bias tool was utilized for evaluating study quality, and statistical analyses were conducted using the RevMan software (Cochrane Collaboration, London, UK).

Patients receiving UDCA demonstrated a significantly lower overall incidence of gallstones post-bariatric surgery (risk ratio [RR] 0.13; *P* < 0.0001). Subgroup analyses confirmed reduced gallstone incidence at three months (*P* = 0.04), six months (*P* < 0.00001), and one year (*P* < 0.00001) with UDCA prophylaxis. Symptomatic cholelithiasis incidence was also lower in the UDCA group (RR 5.70; *P* < 0.00001), and cholecystectomy rates were significantly reduced (RR 3.05; *P* = 0.002).

This meta-analysis supports the efficacy of UDCA prophylaxis in preventing gallstone formation after bariatric surgery. The findings suggest that UDCA administration not only lowers overall gallstone incidence but also reduces the occurrence of symptomatic cholelithiasis and mitigates the need for cholecystectomy. However, caution is warranted due to heterogeneity, diverse surgical procedures, and limited long-term follow-up in the included studies. Further research with standardized protocols and extended observational periods is recommended to strengthen the evidence base and guide clinical practice.

## Introduction and background

Gallstone disease remains a global problem, and it is a common indication for surgical intervention. The prevalence of gallstone disease varies widely among different populations, but the prevalence of 5.9% to 21.9% is commonly reported [1-4].

There are multiple risk factors for gallstones, but the main risk factor associated with the development of this disease is obesity. With the rising prevalence of overweight and obesity around the world, the prevalence of gallstone disease has continued to increase [5-8]. Another risk factor for gallstone formation is rapid weight loss. Rapid weight loss is defined as a reduction of ≥1.5 kg of weight per week, and it causes gallstone formation due to the accelerated elimination of cholesterol, which supersaturates the bile [9-11].

Bariatric surgery is an acceptable treatment for obesity because it causes significant weight loss and resolution of comorbidities. The rapid weight loss following bariatric surgery puts the patients at risk of gallstone formation, which can be a crucial complication [12]. Another reason for increased gallstone formation after bariatric surgery is intestinal dysfunction, leading to decreased cholecystokinin levels, which may cause gallbladder contractile dysfunction [13]. Cholecystectomy is harder after bariatric surgery because it predisposes more postoperative morbidities [12-16].

There are efforts to prevent gallstone disease and its complications after bariatric surgery. Such efforts include prophylactic cholecystectomy during bariatric surgery [14,17,18]. Others include the administration of ursodeoxycholic acid (UDCA) after the surgery to reduce the risk of gallstone formation and cholecystectomy with its attending complications [19-21]. There have been studies assessing the role of UDCA prophylaxis in preventing gallstone formation with conflicting results. Randomized controlled trials (RCTs) have also been conducted to assess the role with some inconsistencies in the results [22-24].

In this systematic review and meta-analysis of RCTs, we aim to assess the role of UDCA prophylaxis in preventing gallstone formation after bariatric surgery.

## Review

Methodology

This systematic review was performed in compliance with the Preferred Reporting Items for Systematic Reviews and Meta-Analyses (PRISMA) guidelines.

Search Strategy

Two independent researchers thoroughly searched the literature across the following databases: PubMed (Medline), Cochrane Central Register of Controlled Studies (CENTRAL), clinical trial registry, ResearchGate, Google Scholar, and Scopus (Elsevier) databases. The last search was conducted in September 2023. The search terms used were “ursodeoxycholic acid,” “Roux-en-Y gastric bypass,” “bariatric surgery,” “sleeve gastrectomy,” “RYGB,” “cholelithiasis,” “SG,” “gallbladder complication,” “UDCA,” “gallbladder disease,” “gallbladder stones,” and “gallstones.” The terms were combined using Boolean operators (AND/OR). Related articles and reference lists were searched to complement the results. Conflicts were resolved by involving a third author.

Study Selection Criteria

The inclusion criteria for a study to be included for the review were as follows: studies published from 1990 to date, RCTs comparing the incidence of gallstone disease post-bariatric surgery in patients who received UDCA and those who did not receive UDCA, studies with full texts, and studies excluding patients with gallstone at the time of bariatric surgery. Conversely, exclusion criteria encompassed any literature reviews, conference presentations, editorials, and commentaries. Also, studies lacking any relevant data for comparison and those with a total study sample size of less than 10 were excluded. Any studies with patients who underwent previous cholecystectomy or gallbladder removal during bariatric surgery were excluded.

Quality Assessment and Risk-of-Bias Assessment

We used the Cochrane Collaboration tool for the risk-of-bias assessment of the included RCTs.

Publication Bias

If 10 or more studies were included in the meta-analysis of a particular outcome, then publication bias was evaluated using the funnel plot and Egger’s test as recommended by the Cochrane handbook.

Data Extraction

Data extraction was performed by two independent authors. The following information was extracted from each study: first author name, year of manuscript publication, study design, number of patients in each group, gender of patients per group, mean age, preoperative body mass index (BMI), the incidence of cases presented with gallstones, cases that had cholecystectomy, and outcome data. In case of conflicts between the two authors, a third author was involved to resolve the conflict.

Outcomes

The primary outcome of interest was the overall incidence of cholelithiasis after bariatric surgery. Secondary outcomes of interest included the incidence of gallstones at three months, six months, and one year after bariatric surgery. Other outcomes of interest included the incidence of symptomatic gallstones and rates of cholecystectomy after bariatric surgery.

Statistical Analysis

Statistical analyses were conducted using RevMan software (version 5.4.1, Cochrane Collaboration, London, UK). If the variable was dichotomous, the pooled odds ratio (OR) was calculated with 95 per cent confidence interval. However, if the variable was continuous, the weighted mean difference (WMD) or standardized mean difference (SMD) with 95% confidence interval (CI) was calculated. Fixed-effects model was used to calculate the pooled effect sizes if the data were not significantly heterogeneous. Otherwise, a random-effects model was used. Heterogeneity was assessed using the I2 statistics. An *I*^2^ value greater than 50% and a *P*-value less than 0.1 were considered indicative of statistically significant heterogeneity. Sensitivity analysis was conducted by sequential elimination of each of the included studies in the meta-analysis to identify the main source of heterogeneity. Publication bias was evaluated using the funnel plot and Egger’s test if 10 or more studies were included in the meta-analysis of a particular outcome as recommended by the Cochrane handbook [25].

Results

Results were reported in accordance with the PRISMA checklist.

Study Selection Process and Description of Selected Studies

**Figure 1:**
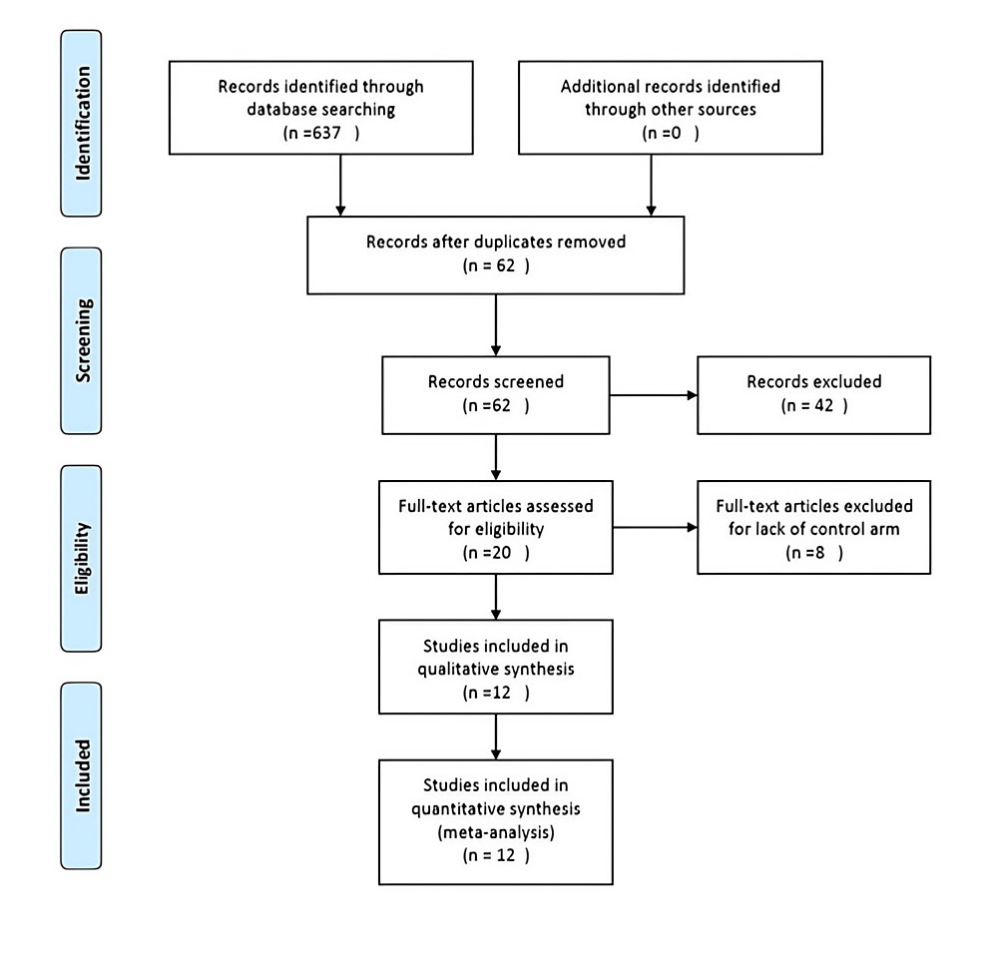
Study selection process.

We identified 637 references during the initial search. Out of these, 575 articles were excluded because of duplicate publications (Figure 1). The other 62 articles underwent title/abstract screening, in which, 42 references were excluded for lack of relevant data. Twenty full-text articles were retrieved, of which eight were excluded for lack of control arm. Twelve studies were included for data synthesis and meta-analysis. The included studies were all RCTs published between 1993 and 2022. Details of the selected studies are displayed in Table 1.

**Table 1:** Characteristics of included studies. UDCA, ursodeoxycholic acid

S/N	Author	Year of publication	Sample size per group		Jadad score	Quality of the study
			UDCA	Placebo		
1	Wudel et al. [22]	2002	13	11	3	Good quality
2	Worobetz et al. [23]	1995	10	14	4	Good quality
3	Talha et al. [24]	2019	1137	295	5	Good quality
4	Adams et al. [26]	2015	37	38	5	Good quality
5	Abouzeid and Shoka [27]	2018	44	45	3	Good quality
6	Pizza et al. [28]	2020	95	95	5	Good quality
7	Sugerman et al. [29]	1995	177	56	3	Good quality
8	Miller et al. [30]	2003	64	60	4	Good quality
9	Tamer et al. [31]	2019	100	100	4	Good quality
10	Sakran et al. [32]	2020	46	46	5	Good quality
11	Williams et al. [33]	1993	44	42	3	Good quality
12	Salman et al. [34]	2022	130	128	4	Good quality

Sociodemographic Variables

No significant difference was found between the two study groups in terms of age distribution (SMD = 0.23; *P* = 0.82), gender distribution (RR = 0.51; *P* = 0.61) and baseline BMI (SMD = 0.91; *P* = 0.36).

Primary outcomes

Gallstones After Bariatric Surgery

**Figure 2:**
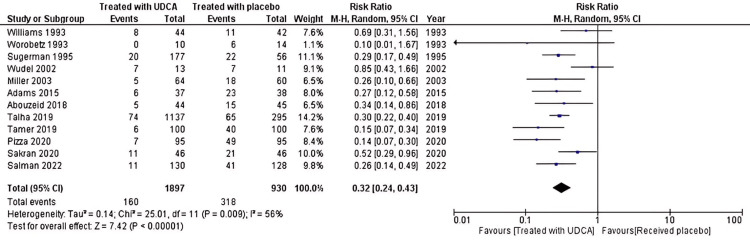
Meta-analysis of the overall incidence of gallstones after bariatric surgery. Wudel et al. [22], Worobetz et al. [23], Talha et al. [24], Adams et al. [26], Abouzeid and Shoka [27], Pizza et al. [28], Sugerman et al. [29], Miller et al. [30], Tamer et al. [31], Sakran et al. [32], Williams et al. [33], Salman et al. [34] UDCA, ursodeoxycholic acid; CI, confidence interval

**Figure 3:**
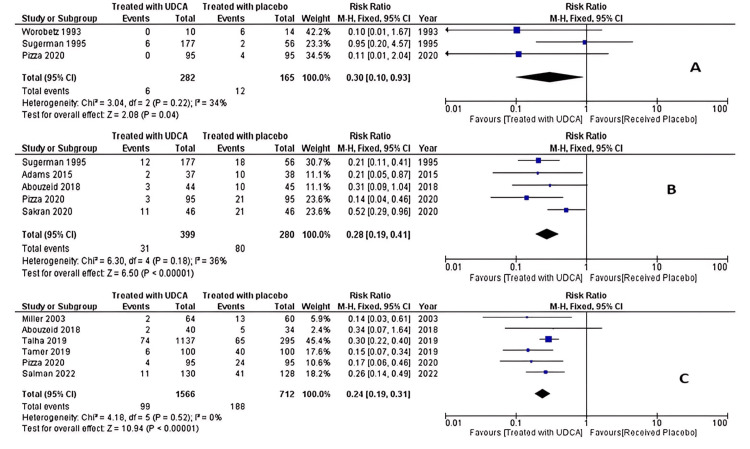
Meta-analysis of incidence of gallstones after bariatric surgery at (A) three months, (B) six months, and (C) one year after surgery. Worobetz et al. [23], Sugerman et al. [29], Pizza et al. [28]. Sugerman et al. [29], Adams et al. [26], Abouzeid and Shoka [27], Pizza et al. [28], Sakran et al. [32]. Miller et al. [30], Abouzeid and Shoka [27], Talha et al. [24], Tamer et al. [31], Pizza et al. [28], Salman et al. [34]. UDCA, ursodeoxycholic acid; CI, confidence interval

The primary outcome we compared was overall incidence of gallstones after bariatric surgery between patients who received UDCA and those who received placebo after bariatric surgery. Twelve studies consisting of a total of 2,767 compared the incidence of gallstones after bariatric surgery [22-24,26-34]. The meta-analysis of these studies revealed that patients who received UDCA after bariatric surgery have a lower incidence of gallstones after bariatric surgery with a risk ratio (RR) of 0.13 (*P* < 0.0001). There was a significant heterogeneity between studies, with *I*^2^ = 56%, so the random effect was used to estimate pooled effect. A subgroup analysis was performed to assess the incidence of gallstones at three months, six months, and one year after surgery, and we found that the patient who received UDCA after surgery had lower incidence of gallstones at each of these periods (*P* = 0.04, *P* < 0.00001, and *P* < 0.00001, respectively). Figure 2 displays the forest plot of the meta-analysis of overall incidence of gallstones, while Figure 3 displays incidence at various time of follow-up.

Secondary outcome

Symptomatic Cholelithiasis

**Figure 4:**
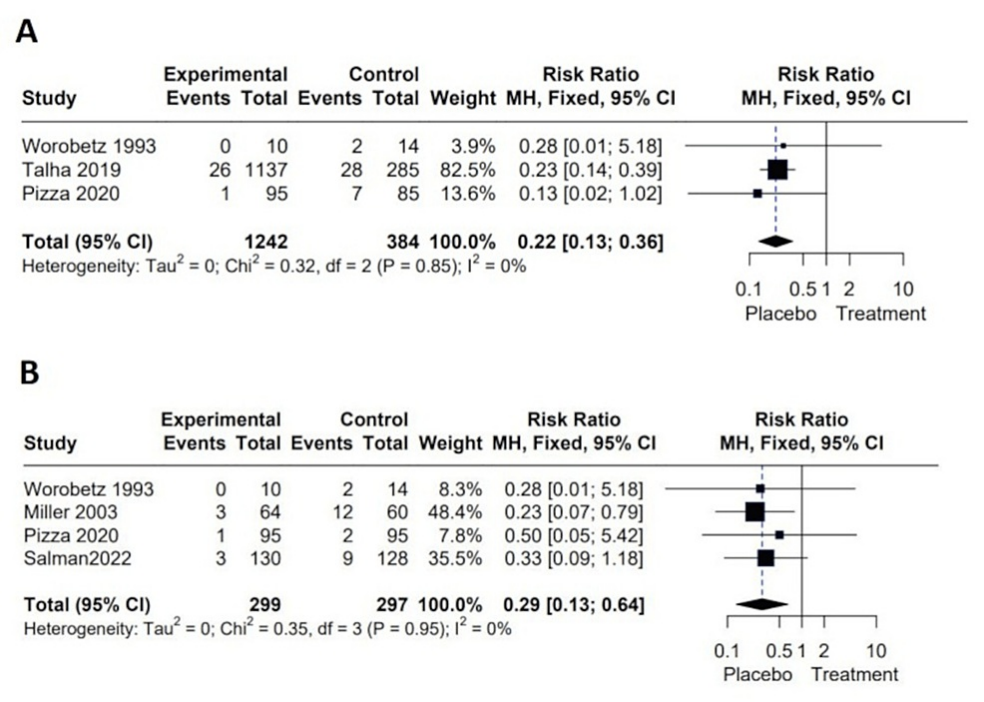
Meta-analysis of the incidence of (A) symptomatic gallstones after bariatric surgery and (B) the rate of cholecystectomy after surgery. Worobetz et al. [23], Talha et al. [24], Pizza et al. [28]. Worobetz et al. [23], Miller et al. [30], Pizza et al. [28], Salman et al. [34].

Three studies consisting of 1,646 patients compared symptomatic cholelithiasis between the two groups [23,24,28]. It was found that patients who received UDCA after surgery tend to have a lesser incidence of symptomatic cholelithiasis than those who received placebo (RR = 5.70; *P* < 0.00001). There was no heterogeneity between the studies as *I*^2^ = 0% (Figure 4A).

Cholecystectomy

Four studies consisting of 596 patients compared cholecystectomy between the two groups [23,28,30,34]. Our meta-analysis revealed that received UDCA after surgery had lesser rate of cholecystectomy compared to those who had placebo with an RR of 3.05 and *P* = 0.002. The heterogeneity between studies was not statistically significant with *I*^2^ = 0% using the fixed effects to estimate pooled effect (Figure 4B).

Discussion

Over the past few decades, it has been observed that obesity is a rising epidemic with reported incidence of up to 30% of the population affected. Bariatric surgery remains the main therapeutic strategy for sustainable weight loss. The rapid weight loss is associated with increased risk of gallstones and its complications such as cholecystitis, cholangitis, or pancreatitis [12,15,17,29,35].

Efforts have been made to prevent gallstones after bariatric surgery, but no consensus has been reached. Prophylactic cholecystectomy has been proposed for prevention, but there are conflicting results regarding increased morbidity or mortality after concomitant cholecystectomy [18,36]. The role of UDCA has also been assessed via multiple RCTs with conflicting results. While some RCTs found that prophylactic administration of UDCA is associated with reduced gall stones [28,29], the RCT conducted by Wudel et al. revealed UDCA has no preventive role [22]. There are some meta-analyses performed to identify the role of UDCA in preventing gallstones after bariatric surgery, but these meta-analyses included retrospective studies, prospective studies, and RCTs [19-21].

In this meta-analysis, we found that prophylactic administration of UDCA after bariatric surgery is associated with reduction in gallstones formation after bariatric surgery. Twelve studies were included in the meta-analysis, and we found significant heterogeneity among the studies. When we did sensitivity analysis, we found that the main source of the heterogeneity was a study by Wudel et al. [22]. When this study was eliminated from the analysis, the UDCA group still had lower incidence of gallstones after surgery. The temporal trend of gallstones was also compared between the two groups, and we found that at three months after bariatric surgery, gallstones are commoner among those who received placebo compared to those who received UDCA. This difference was sustained up to six months and one year after surgery. These findings are similar to findings of previous meta-analysis conducted by Ying et al. [20], Fearon et al. [19], and Magouliotis et al. [21].

We also compared the rate of symptomatic cholelithiasis between the two groups of patients, and we found that symptomatic cholelithiasis is commoner in patients who received placebo compared to those who received UDCA after surgery. This finding is similar to the findings of Haal et al. who reported that UDCA prophylaxis after surgery is associated with reduced incidence of symptomatic cholelithiasis [37].

Cholecystectomy following bariatric surgery is associated with increased morbidity and adverse effect [38,39]. By reducing the rate of gallstones formation and the rate of symptomatic cholelithiasis, it is reported that UDCA prophylaxis after surgery will reduce cholecystectomy among patients who underwent bariatric surgery [23,30,34]. This is what we found in our meta-analysis. In an RCT, Pizza et al. found that UDCA prophylaxis does not affect cholecystectomy despite the fact that patients who received UDCA tended to experience fewer symptoms compared to placebo [28]. This is because some patients in the study refused cholecystectomy after counselling [28].

The findings presented in this meta-analysis on the role of UDCA prophylaxis in preventing gallstone formation after bariatric surgery should be interpreted considering several inherent limitations. First, the included studies exhibited heterogeneity in designs, surgical procedures, and UDCA regimens, potentially influencing the overall outcomes. Moreover, the limited follow-up duration of up to one year might not fully capture long-term trends in gallstone formation post-surgery. Patient heterogeneity, variations in baseline characteristics, and the potential for selective reporting and publication bias further temper the generalizability of the findings. Additionally, the evolution of surgical techniques over time and the geographical specificity of the included studies underscore the need for caution when extrapolating these results to broader populations. Despite these limitations, this meta-analysis offers valuable insights into the potential benefits of UDCA in mitigating gallstone-related complications post-bariatric surgery, prompting the need for further research with standardized protocols and extended follow-up periods to strengthen the evidence base.

## Conclusions

This study reveals that in patients without preoperative gallstones, UDCA prophylaxis can prevent the formation of gallstones after bariatric surgery. In addition, it can significantly reduce the occurrence of symptomatic gallstones and the need for cholecystectomy after bariatric surgery, emphasizing its potential role in minimizing gallstone-related complications. While acknowledging study heterogeneity and limitations, this meta-analysis underscores the importance of further research with standardized protocols and extended follow-up to enhance the robustness of the evidence and inform clinical practice.
